# Sensor-Based Analysis of the Influence of Score Status and Playing Position on the Most Demanding Passages in Elite Women’s Football

**DOI:** 10.3390/s26082349

**Published:** 2026-04-10

**Authors:** Baris Karakoc, Alper Asci, Paweł Chmura

**Affiliations:** 1 Faculty of Sport Sciences, Halic University, Istanbul 34060, Türkiye; bariskarakoc@halic.edu.tr (B.K.); alperasci@halic.edu.tr (A.A.); 2 Department of Individual and Team Sports, Wroclaw University of Health and Sport Sciences, St. I.J. Paderewskiego 35, 51-612 Wrocław, Poland; 3 Department of Sports Science and Clinical Biomechanics, Sport and Health Sciences Cluster (SHSC), Faculty of Health Sciences, University of Southern Denmark, 5230 Odense, Denmark

**Keywords:** worst-case scenario, peak demands, contextual variables, time-series segmentation, fixed-length epoch, wearable inertial sensors

## Abstract

This study aimed to investigate how score status and playing position affect the most demanding passages (MDPs) in elite women’s football. Data from ten matches from eighteen outfield players of the Turkish Women’s National Team were collected during UEFA Nations League fixtures in the 2024–2025 seasons. Players were monitored using wearable GPS sensors, and all locomotor variables were segmented into one-minute windows to identify peak demands. The analysed variables included total distance (TD), high-speed running (HSR), sprint distance (SD), high-acceleration distance (HIAccD), high-deceleration distance (HIDecD), high metabolic power distance (HMPD), and player load (PL). Generalised Estimating Equations (GEE) were used to assess the effects of score status and playing position. Wingers (WG) showed the highest TD, HSR, and HMPD values, while centre backs covered less TD and HSR than WG. Full-backs and forwards (FW) also recorded lower TD, although FW exceeded WG in sprinting (*p* = 0.045, d values = 0.66 [moderate effect]). Score status influenced MDPs, with TD decreasing when the match was tied and further declining when the team was behind; similar reductions occurred in HSR, HIAccD, HIDecD, and HMPD. In conclusion, both score status and position significantly shaped peak locomotor and mechanical demands. These findings may inform individualised training, recovery programmes, and score-dependent tactical planning in elite women’s football.

## 1. Introduction

Over the last 10 years, women’s football participation has grown by a third, and by 2026, worldwide governing bodies aim to have 60 million female players. In recent decades, interest in the sport has increased significantly [1,2,3]. Concurrently, as scientific interest in women’s football has grown, researchers have examined issues such as injuries, anthropometrics, physical performance, and the physical demands of the game [1,3,4]. However, female players are included in less than 15% of professional football research, indicating a glaring disparity in the scientific literature. Compared to the literature on male football, there is still a sizable gap despite the growing number of studies. Therefore, as women’s football evolves and becomes more competitive, more gender-specific data is needed to better understand the factors that affect performance and the sport’s physical demands [1,4,5]. Athletes are subjected to significant physiological and mechanical stress when they engage in high-intensity exercise for more than 90 min with brief rest intervals. Planning and implementing appropriate training tactics that mirror the physical demands of match play is essential to addressing this, especially in the later phases of the game [2,6,7].

Match performance analyses indicate that women cover 9–11 km total distance (TD), with high-speed running distance (HSRD) accounting for ~7.5% of TD. Furthermore, game speed and intensity have increased across three consecutive (2015, 2019, 2023) Women’s World Cups [2,3]. Differences are also observed between national and international competitions; players cover more TD and perform high-speed runs 26% more frequently in international matches [8]. Similar findings indicate that elite female players outperform lower-level peers by ~28% in high-speed running [9]. Acceleration (Acc) and deceleration (Dec) frequencies further reflect match intensity, with women achieving approximately 200–400 high-intensity Acc and Dec per match, depending on the threshold (>2 or >3 m/s^2^) [8]. Additional metrics, including high acceleration distance (HIAccD), high deceleration distance (HIDecD), and player load (PL), provide complementary insights into player performance by capturing the mechanical stresses imposed by rapid changes in speed and direction [2,10].

Monitoring training and match loads is essential for planning training content in accordance with match schedules [11,12]. Recently, the Most Demanding Passages (MDP) method has gained interest for evaluating match load [12,13,14,15]. Traditional average-based analyses underestimate peak demands, potentially leaving players underprepared for high-intensity periods [12,16]. Therefore, MDP analysis provides a more ecologically valid representation of the most intense match scenarios by capturing transient peak demands that are masked by whole-match averages. From a practical perspective, integrating MDP-derived thresholds into training design allows practitioners to replicate worst-case scenarios, optimize conditioning drills, and better prepare players for the most physically demanding phases of competition [17,18].

Comparisons of 90 min averages with one-minute peak values (1 min peak) demonstrate that average match values correspond to only ~60%, ~20%, and ~10% of peak TD, high-speed running, sprinting, and Acc/Dec metrics, respectively [6]. These findings indicate that relying solely on average values may substantially underestimate the intensity of the most demanding phases of match play, thereby limiting the effectiveness of training prescriptions aimed at preparing players for these peak demands. Recent advances in wearable GPS sensor technology have enabled the precise, continuous tracking of players’ movements, accelerations, and mechanical loads in real-time. Modern devices provide higher sampling frequencies (10–20 Hz), improved positioning accuracy, and integrated inertial measurement units (IMUs), allowing for the detailed quantification of high-intensity efforts and locomotor demands. This technological progress facilitates methods such as MDP analysis, enabling researchers and coaches to identify the most physically demanding passages of matches, tailor individualised training programs, and adapt tactical strategies dynamically. Within this framework, wearable sensor technology provides an opportunity to investigate contextual factors that may influence peak locomotor demands during competition. In particular, variables such as playing position and score status may influence how players respond physically to tactical and situational demands throughout a match.

The literature review reveals that MDPs have been investigated under various terms, including ‘most demanding passage’ (MDP), ‘most intense period’ (MIP), and ‘worst-case scenario’ (WCS) [14,16,19,20,21,22]. Studies have examined MDP in terms of time windows [6,18,19], playing positions [18,19], game formation [23], and halves of the game [17], as well as ball possession phases [23,24]. However, no study has examined score status-dependent MDPs in women’s football using wearable sensor technology, which represents a significant gap. Previous research comparing match outcomes (win, draw, loss) found limited differences in physical variables [3,25], but in-game score status within the same match has not been considered. Considering that tactical behaviour and match dynamics often change depending on whether a team is leading, tied, or trailing, examining these contextual scenarios may provide a more ecologically valid understanding of peak match demands. For this reason, the present study had two objectives: first, to compare MDP across different playing positions; second, to evaluate MDP in different score statuses (ahead, tied, behind). Based on the positional differences in physical demands previously reported in football and the tactical adaptations associated with score status, it was hypothesised that (i) MDP values would differ across playing positions and (ii) peak locomotor demands would vary according to score status during the match. This information can support training session planning and provide coaching staff with novel insights for developing tactical and performance strategies.

## 2. Materials and Methods

### 2.1. Study Design

**Figure 1:**
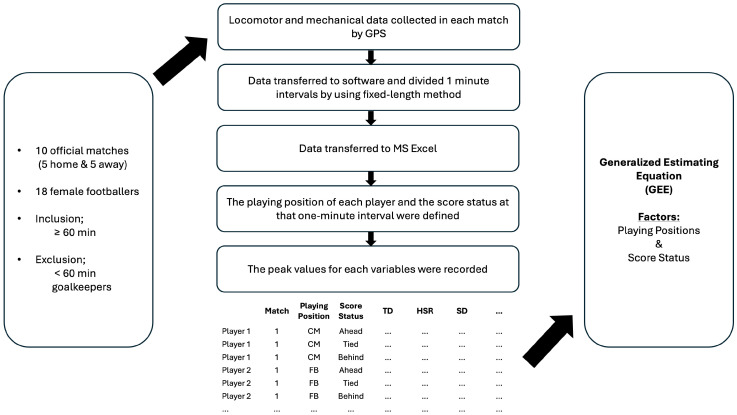
Schematic representation of the study design.

The present study analyses the ten official matches (five home and five away games) played by the Turkish Women’s National Football Team during the 2024–2025 season of the UEFA Women’s Nations League. The analysed matches corresponded to the group stage of the competition, where teams compete to secure promotion or avoid relegation, which may influence the physical and tactical demands of match play. Additionally, the opposing teams represented different competitive standards, as reflected in their international rankings, potentially affecting match intensity and contextual demands. Players’ locomotor and mechanical demands were monitored using wearable GPS devices, enabling the identification of the most demanding passages (MDP) throughout the matches. The use of wearable GPS technology allowed fine-grained, time-resolved assessment of locomotor and mechanical demands, providing detailed insights into peak performance periods. Across the analysed matches, the team consistently adopted a 4–4–2 tactical formation. The necessary permissions for the study were obtained from the Non-Interventional Clinical Research Ethics Committee of the Halic University (387 dated 31 July 2025). Figure 1 shows the study design.

### 2.2. Participants

The present study comprised 18 female footballers (mean age: 27.3 ± 3.4 years; height: 168.6 ± 6.0 cm; body weight: 59.1 ± 4.0 kg) who were members of the Turkish Women’s National Football Team. Because the data were gathered from players on a single national team based on their availability during official matches, the sample was non-probabilistic and based on convenience sampling. As a result, the sample shouldn’t be considered representative of all top women’s football players. Consequently, the findings should be interpreted within the context of this specific team and competition environment. The players were categorised by position on the field: centre back (CB, *n* = 4), fullback (FB, *n* = 4), central midfielder (CM, *n* = 2), winger (WG, *n* = 4), and forward (FW, *n* = 4). The exclusion criteria were: (i) participants had injuries during the study period or within the month preceding the study, (ii) participants did not participate in a minimum of 60 min of play per match, and (iii) participants were not players (goalkeepers). All participants provided informed consent and were informed about the use of wearable GPS devices for data collection.

### 2.3. Procedure

All players were equipped with K-Sports K-50 Live GPS units (K-Sports Tech, Montelabbate, Italy; 10 Hz) and positioned them within vests worn beneath their jerseys. The validity and reliability of these devices have been demonstrated in previous studies [26,27]. Typical measurement errors have been reported to be below ~7.6% for speed-related variables when compared with radar-based reference systems, while coefficients of variation are generally <5–6%, and inter-unit reliability ranges from moderate to excellent (ICC ≈ 0.65–0.99) [28]. Units were activated 15 min prior to each match to ensure a stable satellite connection, and each player used a dedicated unit to prevent inter-unit errors.

Following each match, raw data were exported to K-Fitness software (v. 1.0.3.227, K-Sports Tech, Italy) for processing. To identify the MDPs, a sequential approach was adopted: (1) one-minute fixed-length segments (epochs), which used in previous studies [29,30], were extracted within the software; (2) these locomotor and mechanical responses were transferred to MS Excel, where each epoch was annotated according to the team’s live score status (ahead, tied, or behind); (3) for every individual player in each match, these epochs were grouped by their respective score statuses; and (4) the highest value for each performance variable within each score-status category was identified and extracted as the peak MDP value for that player in that match. A total of 273 one-minute data segments from ten international official matches were included in the final analysis.

### 2.4. Variables

Dependent Variables: The MDP assessment included the following performance variables: total distance (TD), high-speed running distance (HSR, 19–22.9 km/h), sprint distance (SD, ≥23 km/h), high acceleration distance (HIAccD), acceleration (Acc) ≥ 3 m/s^2^), high deceleration distance (HIDecD), deceleration (Dec) ≤ −3 m/s^2^), high metabolic power distance (HMPD), metabolic power ≥ 20 W/kg), and player load (PL) [7,31].

MDP values were identified as the highest for each variable within each score status for each player in each game, during each one-minute segment, and were subsequently used in statistical analyses.

The categorical factors used to explain variations in performance were (explanatory Factors):

Playing Positions: CB, FB, CM, WG, and FW.

Score Status: Ahead, tied, and behind.

### 2.5. Statistical Analysis

The analysis was conducted using a Generalized Estimating Equation (GEE) model, consistent with previous performance analysis studies [32,33]. This approach accounts for the correlated structure of the data arising from repeated measures, including multiple matches per player and one-minute GPS-based segments annotated by score status. This structure was chosen to provide a parsimonious approach for modelling repeated observations while avoiding unnecessary assumptions about the correlation pattern between measurements.

All match performance variables derived from wearable GPS data (TD, HSR, SD, HIAccD, HIDecD, HMPD, PL) were considered as dependent variables. The factors were set as playing positions (CB, FB, CM, WG, FW) and score status (ahead, tied, behind). The GEE models included the main effects of playing position and score status, as well as the interaction term between these two factors (playing position × score status). Subject effects were specified as individual players, and within-subject effects were matches and score status. An identity link function was applied, and the working correlation structure was set as independent.

Wald χ^2^ tests were used to assess the significance of model effects and parameter estimates. Analyses were performed using SPSS (version 24, IBM, Armonk, NY, USA) with a significance threshold of *p* < 0.05. As SPSS does not provide effect sizes directly for GEE results, effect sizes were calculated using the B coefficients (differences between each category and the reference group) obtained from GEE parameter estimates and the overall standard deviation of the variable (d = B/SDgeneral). Effect size magnitudes were classified as trivial (<0.2), small (0.2–0.59), moderate (0.6–1.19), large (1.2–1.99), and very large (≥2.0) [34].

## 3. Results

**Figure 2:**
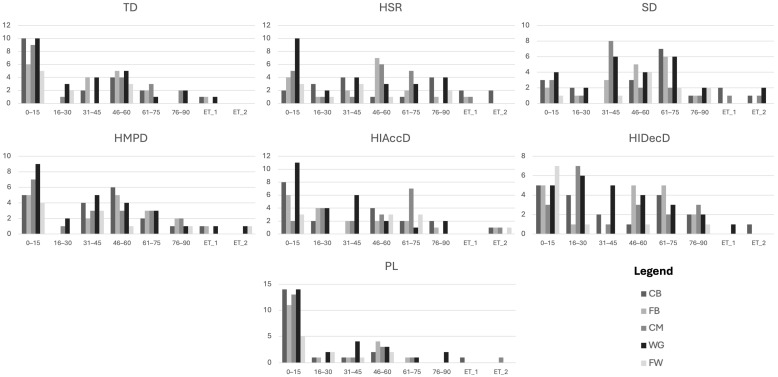
Frequency distribution of MDP for different playing positions through different time periods.

The descriptive statistics for match performance variables by position and score status are shown in Table 1. Frequency distribution graphs are shown in Figure 2, showing the time periods during which players in different positions achieved their MDP. These descriptive outputs provide the contextual foundation for subsequent modelling and facilitate interpretation of between-position and between-status variability typical of applied GPS-based performance monitoring.

Parameter estimates were evaluated relative to reference values based on GEE analysis. The WG category for Position and the Ahead category for Score were taken as references by the software. The ‘B’ values in Table 2 indicate the difference between the relevant category and the reference category. This analytical approach enables quantification of position- and context-specific deviations while accounting for repeated, sensor-derived observations within players.

### 3.1. Position Effect

GEE analysis revealed a significant main effect for position on TD (Wald χ^2^ = 49.49, df = 4, *p* < 0.001). Parameter estimates indicate that CB covered an average distance of 31.24 m less than WG (*p* < 0.001). Similarly, CM (−12.46 m), FW (−25.26 m), and FB (−20.68 m) had significantly lower TD values than WG. Furthermore, a significant main effect was identified in the HSR (Wald χ^2^ = 93.77, df = 4, *p* < 0.001). The parameter estimates demonstrated that CB and FB exhibited significantly lower HSR performance in comparison to WG (−21.08 and −14.85 m, respectively). Conversely, CM (−10.70 m) and FW (−12.97 m) did not demonstrate a significant difference. A significant main effect was identified for SD (Wald χ^2^ = 17.87, df = 4, *p* = 0.001), with CB covering a shorter (−8.17 m; *p* = 0.048) and FW covering a longer sprint distance (8.20 m; *p* = 0.045) compared to WG. No significant differences were identified in the remaining positions (*p* > 0.05). The results revealed no substantial impact of position on HIAccD (Wald χ^2^ = 7.71, df = 4, *p* = 0.103). These findings are also supported by the parameter estimations (*p* > 0.05). The HIDecD variable showed significant main effects for position (Wald χ^2^ = 46.77, df = 4, *p* < 0.001). CB and CM had lower HIDecD than WG (−2.15 m and −1.45 m, respectively), while no significant difference was found for other positions (*p* > 0.05). Significant main effects were found for HMPD (Wald χ^2^ = 78.83, df = 4, *p* < 0.001). CB, FW, and FB showed significantly lower performance compared to WG (−27.90 m, −16.82, and −16.95 m, respectively). Significant results were obtained for the PL variable in the main effect of position (Wald χ^2^ = 11.98, df = 4, *p* = 0.017), while no significant difference was found in the parameter estimates compared to the reference values in different positions.

### 3.2. Score Effect

An examination of the score status in the matches revealed a substantial main effect for TD (Wald χ^2^ = 39.93, df = 2, *p* < 0.001). The parameter estimates indicate that the total distance in the tie is 6.04 m lower than in the sections where the game was ahead (*p* < 0.001), while in the case of being behind, this difference is −20.21 m (*p* < 0.001). There are also significant main effects for HSR (Wald χ^2^ = 6.66, df = 2, *p* = 0.036). Players perform significantly less HSR when their team is behind than when they are ahead (B = −12.05, *p* = 0.001). No significant differences were found in the main effect for SD (Wald χ^2^ = 5.70, df = 2, *p* = 0.058) or in the parameter estimates (*p* > 0.05). The score status for HIAccD revealed a significant main effect (Wald χ^2^ = 39.65, df = 2, *p* < 0.001). Players demonstrated a marked decline in performance when their team was behind compared to when they were ahead (B = −1.14, *p* < 0.001). A significant main effect was found for HIDecD variable (Wald χ^2^ = 21.84, df = 2, *p* < 0.001). According to parameter estimates, the team performs lower HIDecD (−1.93 m, *p* < 0.001) when behind. The score status for HMPD exhibited a significant main effect (Wald χ^2^ = 84.01, df = 2, *p* < 0.001). When the team is behind, the findings are significantly lower than when they are ahead (B = −17.54, *p* < 0.001). In the PL, no significant difference was found either in terms of the main effect (Wald χ^2^ = 5.47, df = 2, *p* = 0.065) or in terms of parameter estimates compared to reference values (*p* > 0.05).

### 3.3. Position × Score Interactions

The interaction results for playing position and score status are shown in Table 3. Since the model selected WG for the playing position and Ahead for the score statuses, the reference value for interaction results was set to WG × Ahead. There are small to large interactions were found for TD (Wald χ^2^ = 36.88, df = 8, *p* < 0.001), HSR (Wald χ^2^ = 18.59, df = 8, *p* < 0.05), HIDecD (Wald χ^2^ = 16.05, df = 8, *p* < 0.05) and PL (Wald χ^2^ = 59.64, df = 8, *p* < 0.001).

## 4. Discussion

The aim of this study was to investigate how different positions and score statuses in the game affect MDP among elite women footballers. Importantly, this study used high-resolution GPS tracking and match analysis to detect subtle position-specific and score-dependent variations in MDP, demonstrating the potential of wearable sensors and advanced data analytics in elite football. With the exception of HIAccD and PL, WGs demonstrated greater values across all match performance variables than CBs. FBs showed the next-highest performance, with significant differences observed in TD, HSR, and HMPD; CMs had higher TD and HIDecD than WGs, and FWs had higher SD than WGs.

Other studies have also indicated that the WG is more physically demanding than other positions. In one such study [16], wide midfielders and FB exhibited higher HSR and SD than players in other positions. Similarly, Thoseby et al. [15] noted that WG have higher HSR than the remaining positions. By contrast, Martín-García et al. [21] found that the WG position produced the lowest SD response, similar to the midfielder position. In the same study, WG was found to be the best-performing position for TD, consistent with our findings. However, these studies have been conducted on men’s football. One of the few studies conducted on women found that WG players produced higher HSR and TD responses than CB [2]. Another study on women footballers observed the highest very HSR in WG and FW, and SD in FB [10]. A further study, which did not examine MDP but investigated physical performance in female players, indicated that wing players covered significantly greater distances than CM and CB players in both very high-speed (>18 km/h) and sprint (>25 km/h) zones [5]. The higher performance demonstrated in TD for WG compared to other positions is consistent with the findings of the two above-mentioned studies [2,18].

The elevated physical demands observed in WGs could be interpreted through the lens of established tactical theories in elite football, as stated in a previous study [2]. While tactical roles were not directly monitored, it is speculative but possible that these demands reflect roles such as supporting central midfielders or covering diagonal passes. These hypothesized roles align with the game’s intermittent profile but require future tactical verification. Their lower SD than FWs could indicate fatigue from sustained ball- and non-ball-possession efforts. However, the tactical structures were not directly measured in the current study, as stated above; previous studies have highlighted the effects of tactical situations on running performance [15]. These interpretations appear consistent with the typical intermittent profile of women’s elite football and may reinforce the position-specific nature of peak demands. Therefore, the present findings suggest that, within similar elite national team contexts, coaches might consider individualizing training and recovery strategies for WG, particularly when planning high-speed and Acc-based training loads.

Few studies have investigated MDP in women’s football; TD and HSR are commonly reported, while SD, HIAccD, HIDecD, HMPD, and PL are less studied. In a study of women footballers using these variables, MDP responses were not evaluated according to playing position [6]. In one of the rare studies to use these variables for MDP, WG, and FW players produced the highest responses for HIAccD and HIDecD. This aligns with the results of our study for HIDecD [10]. The higher HMPD values observed among WG in the current study could indicate a greater exposure to mechanical load, potentially carrying implications for fatigue management and injury risk that warrant further investigation. Although a significant main effect for PL was found in this study, no significant differences were observed between positions. This is thought to be because the difference between the positions is not statistically strong enough. Of the few studies conducted on PL, none found a significant main effect with a 1 min epoch [2,10].

The present study identified significant score-dependent differences, with higher MDP values observed when the team was ahead, indicating greater physical effort [2,25,35]. This increase may potentially coincide with theorized tactical principles where leading teams adopt compact defensive structures to maintain an advantage. While not directly measured in this study, it is hypothesized that such a defensive approach—potentially involving frequent transitions or closing passing lanes—could explain the repeated mechanical efforts observed. In addition to these actions, leading teams can counter-attack by making high-speed runs, especially with WG and FW players [6,23,25]. Furthermore, from a psychological standpoint, players on the trailing team may experience increased motivation and concentration, which can lead to a faster pace of the game, even if only for a short time [35,36]. Additionally, psychological factors, such as shifting motivation levels based on the scoreline, might also contribute to these variations. Coaches operating in similar elite settings might therefore benefit from considering these potential hypothesized tactical and psychological links as a basis for interpreting score-dependent performance outcomes.

Some studies assessed general physical performance based on match results rather than score status, without examining MDP [3]. In a rare study examining peak physical demands in women’s soccer, a main effect of match outcome was observed for TD and HIDecD, similar to the current study [10]. Another study, similar to the present one, examined the effect of the first goal scored or conceded in the MDP (one-minute epoch). It found that the goal effect for HSR was significant, irrespective of whether a goal was scored or conceded [2].

In line with our GPS-based MDP analysis, TD, HSR, HIAccD, HIDecD, and HMPD were higher when the team was ahead than when tied or behind, indicating greater effort during a lead. These observations could potentially align with tactical principles in elite women’s football, where it is hypothesized that teams may adopt a more compact defensive structure when leading. This plausible explanatory pathway, which might involve repeated accelerative efforts for pressing or rapid compacting between lines, illustrates how high-resolution tracking can capture nuanced performance variations; however, these links between locomotor output and tactical behavior remain speculative explanatory pathways requiring future verification with positional data. Future studies should integrate contextual variables such as possession, pressing style, and block height to better understand the mechanisms underlying these score status effects. A valuable study aimed at this purpose compared MDP performance with and without ball possession but did not consider game outcomes or scores [23].

The study has its limitations. Firstly, although the data were collected during international matches, they pertain to a single team and constitute a small dataset. When evaluating the findings, researchers and practitioners should consider key factors such as the team’s positioning on the pitch, the quality of opponents, opponents’ playing style, and opponents’ formation. Consequently, the findings may reflect this team’s particular tactical approach, physical conditioning, and contextual characteristics rather than being representative of all elite women’s football teams. For this reason, they should not be generalised to different teams and tournaments. In addition, the MDP data used in the study were determined using the fixed-length method. Due to limited access to the raw data recorded by the GPS units, it was not possible to apply a moving average using the computer software. Therefore, the method used in this study differs from that used in some other studies in the literature. This methodological constraint should be considered when comparing our results with studies using variable-epoch or moving-average techniques, which typically yield higher peak values. Nonetheless, the consistent application of the same method across all matches in our dataset strengthens the internal validity of the current findings.

## 5. Conclusions

In conclusion, wingers exhibited the highest MDP values, and players exerted more effort when the team was ahead, suggesting the potential benefits of position-specific training and score-status-informed tactical strategies within this competitive framework. These preliminary insights could offer a basis for coaches in similar elite environments to design defensive transition drills, fitness conditioning exercises, and match-preparation drills. Importantly, the use of high-resolution GPS tracking and advanced match analysis technology demonstrates how these tools can capture nuanced, position- and score status-dependent performance patterns through a fixed-length method, supporting evidence-based planning and individualized load management. Furthermore, future research should expand on these findings by investigating MDP across multiple teams and incorporating different phases of play and ball possession situations.

## 6. Practical Implication

Beyond its practical significance, this study contributes to the growing body of literature on the physical and contextual determinants of performance in women’s football, a field that remains underrepresented compared to men’s football. By applying a Generalized Estimating Equation approach, the study accounts for repeated measures and correlated data structures, providing a robust framework for analysing positional and situational influences on MDP. Future research would benefit from integrating larger and more diverse samples across multiple competition levels and from employing dynamic time-window analyses (moving-average approach) to complement fixed-length approaches. Such methodological advancements can improve the validity of MDP assessment and support the development of individualized, evidence-based performance models tailored to the specific demands of elite women’s football in comparable international competition settings.

## Figures and Tables

**Table 1:** The descriptive statistics for match performance variables by position and score status.

			N	Mean	SD	CV (%)	Minimum	Maximum
TD	Position	CB	29	157.62	16.51	10.48	125.0	197.0
		CM	33	179.64	17.03	9.48	142.0	216.0
		FW	19	166.05	19.45	11.71	132.0	215.0
		FB	34	166.50	16.75	10.06	121.0	200.0
		WG	50	180.14	14.48	8.04	153.0	206.0
	Score	Ahead	44	171.57	18.89	11.01	142.0	206.0
		Behind	36	166.08	19.89	11.98	121.0	207.0
		Tied	85	174.05	17.38	9.99	132.0	216.0
HSR	Position	CB	29	27.94	9.69	34.69	12.2	48.4
		CM	33	35.28	10.99	31.15	14.8	61.9
		FW	19	30.83	8.96	29.05	12.8	49.2
		FB	34	27.44	11.19	40.77	10.7	69.4
		WG	50	37.57	13.79	36.71	11.3	74.9
	Score	Ahead	44	32.91	12.84	39.01	12.2	67.9
		Behind	36	31.25	10.40	33.27	11.3	61.9
		Tied	85	32.92	12.68	38.50	10.7	74.9
SD	Position	CB	29	18.95	11.38	60.05	4.5	55.0
		CM	33	18.39	9.93	54.02	0.7	39.7
		FW	19	31.30	12.42	39.67	13.0	60.7
		FB	34	21.70	11.43	52.68	1.9	43.3
		WG	50	22.86	13.73	60.07	0.7	62.2
	Score	Ahead	44	23.55	11.32	48.07	1.3	48.3
		Behind	36	22.33	12.47	55.86	0.7	60.7
		Tied	85	21.08	13.07	62.01	0.7	62.2
HMPD	Position	CB	29	59.74	14.49	24.26	38.6	105.0
		CM	33	80.08	17.77	22.20	53.0	118.3
		FW	19	74.52	16.42	22.04	42.8	117.4
		FB	34	66.94	16.25	24.27	37.0	112.9
		WG	50	81.30	15.88	19.53	52.2	120.9
	Score	Ahead	44	75.38	17.37	23.05	46.0	111.7
		Behind	36	66.62	15.26	22.91	37.0	97.2
		Tied	85	75.50	18.91	25.04	38.8	120.9
HIAccD	Position	CB	29	4.27	1.35	31.52	2.4	8.0
		CM	33	3.48	0.99	28.51	1.4	5.3
		FW	19	4.17	0.93	22.29	2.8	6.0
		FB	34	3.69	0.86	23.32	2.0	6.0
		WG	50	4.09	1.08	26.38	1.1	7.0
	Score	Ahead	44	4.21	1.02	24.15	2.1	6.0
		Behind	36	3.35	1.18	35.21	1.1	6.0
		Tied	85	4.02	1.00	24.89	1.9	8.0
HIDecD	Position	CB	29	4.23	1.00	23.69	2.7	6.0
		CM	33	5.08	0.98	19.21	3.0	7.8
		FW	19	5.62	1.57	27.95	1.6	9.0
		FB	34	5.36	1.20	22.42	2.7	8.0
		WG	50	5.88	1.50	25.54	2.0	9.8
	Score	Ahead	44	5.44	1.61	29.62	2.7	9.8
		Behind	36	4.84	1.37	28.37	1.6	8.0
		Tied	85	5.41	1.23	22.75	2.7	9.0
PL	Position	CB	29	6.41	2.53	39.43	4.0	12.0
		CM	33	10.27	3.63	35.31	5.0	16.0
		FW	19	9.84	3.06	31.09	5.0	17.0
		FB	34	8.38	3.44	40.97	4.0	15.0
		WG	50	9.06	3.24	35.71	5.0	16.0
	Score	Ahead	44	8.36	3.47	41.51	4.0	15.0
		Behind	36	9.25	3.74	40.48	4.0	16.0
		Tied	85	8.81	3.30	37.41	4.0	17.0

SD: Std. Deviation, CV: Coefficient of variation.

**Table 2:** Generalized Estimating Equation Analysis (main effects).

			B	SE	95% CI		Wald χ^2^	*p*		d	
					Lower	Upper					
TD	Position	CB	−31.24	5.45	−41.92	−20.56	32.86	0.000	***	−1.57	large
		CM	−12.46	5.33	−22.90	−2.02	5.47	0.019	*	−0.63	moderate
		FW	−25.26	3.94	−32.98	−17.55	41.19	0.000	***	−1.27	large
		FB	−20.68	4.78	−30.06	−11.31	18.71	0.000	***	−1.04	moderate
	Score	Tied	−6.04	1.51	−9.00	−3.08	15.98	0.000	***	−0.30	small
		Behind	−20.21	3.96	−27.98	−12.45	26.04	0.000	***	−1.02	moderate
HSR	Position	CB	−21.08	5.08	−31.03	−11.13	17.24	0.000	***	−1.65	large
		CM	−10.70	6.01	−22.48	1.07	3.17	0.075		-	-
		FW	−12.97	7.03	−26.75	0.80	3.41	0.065		-	-
		FB	−14.85	3.57	−21.85	−7.86	17.34	0.000	***	−1.16	moderate
	Score	Tied	−6.09	4.34	−14.61	2.42	1.97	0.161		-	-
		Behind	−12.05	3.61	−19.14	−4.97	11.12	0.001	**	−0.94	moderate
SD	Position	CB	−8.17	4.12	−16.26	−0.09	3.93	0.048	*	−0.66	moderate
		CM	−7.31	4.17	−15.48	0.87	3.07	0.080		-	-
		FW	8.20	4.09	0.18	16.21	4.02	0.045	*	0.66	moderate
		FB	1.55	4.39	−7.06	10.16	0.13	0.724		-	-
	Score	Tied	−3.27	1.87	−6.94	0.40	3.06	0.080		-	-
		Behind	−4.18	3.12	−10.29	1.94	1.79	0.181		-	-
HIAccD	Position	CB	−0.27	0.57	−1.39	0.85	0.22	0.636		-	-
		CM	−0.47	0.56	−1.57	0.64	0.69	0.405		-	-
		FW	0.25	0.64	−1.01	1.51	0.15	0.697		-	-
		FB	−0.26	0.38	−1.01	0.50	0.45	0.502		-	-
	Score	Tied	−0.10	0.30	−0.69	0.49	0.11	0.740		-	-
		Behind	−1.14	0.30	−1.72	−0.55	14.38	0.000	***	−1.02	moderate
HIDecD	Position	CB	−2.15	0.47	−3.07	−1.23	20.96	0.000	***	−1.44	large
		CM	−1.45	0.43	−2.29	−0.62	11.62	0.001	**	−0.97	moderate
		FW	−0.74	0.93	−2.56	1.08	0.64	0.424		-	-
		FB	−0.72	0.58	−1.85	0.42	1.53	0.216		-	-
	Score	Tied	−0.21	0.39	−0.98	0.56	0.29	0.591		-	-
		Behind	−1.93	0.54	−2.98	−0.88	12.97	0.000	***	−1.29	large
HMPD	Position	CB	−27.90	5.61	−38.90	−16.91	24.73	0.000	***	−1.51	large
		CM	−9.27	5.45	−19.95	1.42	2.89	0.089		-	-
		FW	−16.82	6.08	−28.72	−4.91	7.66	0.006	**	−0.91	moderate
		FB	−16.95	4.96	−26.67	−7.23	11.67	0.001	**	−0.92	moderate
	Score	Tied	−5.60	3.33	−12.12	0.93	2.83	0.093		-	-
		Behind	−17.54	4.06	−25.49	−9.59	18.69	0.000	***	−0.95	moderate
PL	Position	CB	−2.33	1.43	−5.14	0.49	2.63	0.105		-	-
		CM	−0.17	1.41	−2.92	2.59	0.02	0.904		-	-
		FW	0.23	1.37	−2.45	2.91	0.03	0.866		-	-
		FB	0.23	1.65	−3.00	3.46	0.02	0.888		-	-
	Score	Tied	0.31	0.50	−0.68	1.30	0.37	0.542		-	-
		Behind	0.31	0.93	−1.51	2.13	0.11	0.735		-	-

SE: Standard error, d: Effect size * *p* < 0.05, ** *p* < 0.01, *** *p* < 0.001.

**Table 3:** Generalized Estimating Equation Analysis (position × score interaction).

			B	SE	95% CI		Wald χ^2^	*p*		d	
					Lower	Upper					
TD	Position × Score	CB × Tied	9.71	3.59	2.67	16.74	7.30	0.007	**	0.49	small
		CB × Behind	14.63	6.71	1.48	27.77	4.76	0.029	*	0.74	moderate
		CM × Tied	10.25	4.03	2.35	18.15	6.46	0.011	*	0.52	small
		CM × Behind	12.21	3.98	4.41	20.01	9.41	0.002	**	0.61	moderate
		FW × Tied	14.84	5.05	4.94	24.74	8.63	0.003	**	0.75	moderate
		FW × Behind	8.01	8.34	−8.33	24.36	0.92	0.337		-	-
		FB × Tied	8.32	3.06	2.32	14.31	7.39	0.007	**	0.42	small
		FB × Behind	9.89	7.49	−4.78	24.56	1.74	0.187		-	-
HSR	Position × Score	CB × Tied	9.52	5.92	−2.09	21.13	2.58	0.108		-	-
		CB × Behind	17.04	4.54	8.15	25.93	14.10	0.000	***	1.33	large
		CM × Tied	7.38	4.99	−2.40	17.17	2.19	0.139		-	-
		CM × Behind	10.38	8.10	−5.49	26.25	1.64	0.200		-	-
		FW × Tied	7.83	7.41	−6.69	22.35	1.12	0.290		-	-
		FW × Behind	5.39	7.43	−9.18	19.97	0.53	0.468		-	-
		FB × Tied	5.30	5.90	−6.27	16.87	0.81	0.370		-	-
		FB × Behind	4.34	4.56	−4.60	13.29	0.91	0.341		-	-
SD	Position × Score	CB × Tied	5.57	3.91	−2.08	13.23	2.04	0.154		-	-
		CB × Behind	6.11	3.97	−1.67	13.89	2.37	0.124		-	-
		CM × Tied	4.40	2.32	−0.15	8.96	3.59	0.058		-	-
		CM × Behind	1.21	3.87	−6.38	8.80	0.10	0.755		-	-
		FW × Tied	−2.76	2.89	−8.41	2.90	0.91	0.339		-	-
		FW × Behind	7.95	7.02	−5.82	21.71	1.28	0.258		-	-
		FB × Tied	−5.16	3.63	−12.28	1.96	2.02	0.155		-	-
		FB × Behind	0.45	5.45	−10.23	11.13	0.01	0.934		-	-
HIAccD	Position × Score	CB × Tied	0.47	0.38	−0.26	1.21	1.59	0.208		-	-
		CB × Behind	0.76	0.43	−0.08	1.61	3.15	0.076		-	-
		CM × Tied	−0.40	0.31	−1.00	0.20	1.71	0.190		-	-
		CM × Behind	−0.17	0.33	−0.82	0.47	0.28	0.597		-	-
		FW × Tied	−0.49	0.54	−1.55	0.57	0.82	0.366		-	-
		FW × Behind	0.48	0.64	−0.77	1.73	0.56	0.456		-	-
		FB × Tied	−0.23	0.34	−0.89	0.42	0.48	0.487		-	-
		FB × Behind	−0.01	0.37	−0.74	0.71	0.00	0.975		-	-
HIDecD	Position × Score	CB × Tied	0.39	0.44	−0.47	1.25	0.79	0.373		-	-
		CB × Behind	1.26	0.71	−0.12	2.65	3.19	0.074		-	-
		CM × Tied	0.13	0.52	−0.90	1.16	0.06	0.807		-	-
		CM × Behind	1.52	0.69	0.17	2.86	4.91	0.027	*	1.02	moderate
		FW × Tied	0.62	0.87	−1.08	2.33	0.51	0.475		-	-
		FW × Behind	0.57	1.03	−1.45	2.59	0.30	0.582		-	-
		FB × Tied	−0.36	0.52	−1.39	0.66	0.48	0.490		-	-
		FB × Behind	1.36	0.64	0.10	2.62	4.45	0.035	*	0.91	moderate
HMPD	Position × Score	CB × Tied	6.11	4.86	−3.41	15.63	1.58	0.208		-	-
		CB × Behind	11.54	5.91	−0.04	23.12	3.81	0.051		-	-
		CM × Tied	6.35	5.98	−5.37	18.08	1.13	0.288		-	-
		CM × Behind	7.82	4.92	−1.82	17.46	2.53	0.112		-	-
		FW × Tied	12.09	7.67	−2.95	27.12	2.48	0.115		-	-
		FW × Behind	11.92	9.48	−6.65	30.49	1.58	0.208		-	-
		FB × Tied	3.01	4.39	−5.59	11.61	0.47	0.492		-	-
		FB × Behind	2.08	6.94	−11.51	15.68	0.09	0.764		-	-
PL	Position × Score	CB × Tied	−0.20	0.60	−1.38	0.99	0.11	0.745		-	-
		CB × Behind	−0.58	0.97	−2.47	1.32	0.36	0.551		-	-
		CM × Tied	1.30	0.69	−0.05	2.66	3.56	0.059		-	-
		CM × Behind	1.99	0.88	0.27	3.70	5.15	0.023	*	0.58	small
		FW × Tied	0.69	0.95	−1.17	2.55	0.53	0.466		-	-
		FW × Behind	−0.11	1.57	−3.18	2.95	0.01	0.942		-	-
		FB × Tied	−1.03	0.68	−2.36	0.30	2.31	0.128		-	-
		FB × Behind	−1.86	1.01	−3.84	0.12	3.39	0.065		-	-

SE: Standard error, d: Effect size * *p* < 0.05, ** *p* < 0.01, *** *p* < 0.001.

## Data Availability

The raw data supporting the conclusions of this article will be made available by the authors on request.

## Abbreviations

The following abbreviations are used in this manuscript:

MDP	Most Demanding Passages
GPS	Global Positioning System
TD	Total Distance
HSR	High-Speed Running
SD	Sprint Distance
HIAccD	High-Acceleration Distance
HIDecD	High-Deceleration Distance
HMPD	High Metabolic Power Distance
PL	Player Load
CB	Centre Back
FB	Fullback
CM	Central Midfielder
WG	Winger
FW	Forward
GEE	Generalized Estimating Equation
